# Assessment of *k*-mer spectrum applicability for metagenomic dissimilarity analysis

**DOI:** 10.1186/s12859-015-0875-7

**Published:** 2016-01-16

**Authors:** Veronika B. Dubinkina, Dmitry S. Ischenko, Vladimir I. Ulyantsev, Alexander V. Tyakht, Dmitry G. Alexeev

**Affiliations:** Research Institute of Physico-Chemical Medicine, Malaya Pirogovskaya, Moscow, 119435 Russia; Moscow Institute of Physics and Technology (State University), Institutskiy per., Dolgoprudny, 141700 Russia; ITMO University, Kronverksky Pr., St. Petersburg, 197101 Russia

**Keywords:** *k-*mer, *n-*gram, *l-*tuple, Sequence signature, Gut metagenome, Phage, Reference-free metagenomic analysis, Genomic variability, Mapping bias

## Abstract

**Background:**

A rapidly increasing flow of genomic data requires the development of efficient methods for obtaining its compact representation. Feature extraction facilitates classification, clustering and model analysis for testing and refining biological hypotheses. “Shotgun” metagenome is an analytically challenging type of genomic data - containing sequences of all genes from the totality of a complex microbial community. Recently, researchers started to analyze metagenomes using reference-free methods based on the analysis of oligonucleotides (*k-*mers) frequency spectrum previously applied to isolated genomes. However, little is known about their correlation with the existing approaches for metagenomic feature extraction, as well as the limits of applicability. Here we evaluated a metagenomic pairwise dissimilarity measure based on short *k-*mer spectrum using the example of human gut microbiota, a biomedically significant object of study.

**Results:**

We developed a method for calculating pairwise dissimilarity (beta-diversity) of “shotgun” metagenomes based on short *k-*mer spectra (5≤*k*≤11). The method was validated on simulated metagenomes and further applied to a large collection of human gut metagenomes from the populations of the world (*n*=281). The *k-*mer spectrum-based measure was found to behave similarly to one based on mapping to a reference gene catalog, but different from one using a genome catalog. This difference turned out to be associated with a significant presence of viral reads in a number of metagenomes. Simulations showed limited impact of bacterial genetic variability as well as sequencing errors on *k-*mer spectra. Specific differences between the datasets from individual populations were identified.

**Conclusions:**

Our approach allows rapid estimation of pairwise dissimilarity between metagenomes. Though we applied this technique to gut microbiota, it should be useful for arbitrary metagenomes, even metagenomes with novel microbiota. Dissimilarity measure based on *k-*mer spectrum provides a wider perspective in comparison with the ones based on the alignment against reference sequence sets. It helps not to miss possible outstanding features of metagenomic composition, particularly related to the presence of an unknown bacteria, virus or eukaryote, as well as to technical artifacts (sample contamination, reads of non-biological origin, etc.) at the early stages of bioinformatic analysis. Our method is complementary to reference-based approaches and can be easily integrated into metagenomic analysis pipelines.

**Electronic supplementary material:**

The online version of this article (doi:10.1186/s12859-015-0875-7) contains supplementary material, which is available to authorized users.

## Background

During the last decade, metagenomics became one of the explosively developing areas of molecular genomics. Advent of the next-generation sequencing allowed performing genomic analysis of samples obtained directly from the environment. Such an approach provides data for an extensive quantitative examination of the microbial community structure, particularly including uncultivable and previously undiscovered components. The sphere of metagenomic analysis has extended from science to heavy industry [1], agriculture [2, 3] and healthcare [4]. A large amount of metagenomic data is constantly being accumulated which leads to a demand in the means of efficient analysis [5].

One of the common steps in metagenomic study is calculation of pairwise dissimilarity between the samples (beta-diversity) [6]. Beta-diversity is a quantitative measure of the differences in composition between two microbial communities. Its value is calculated from features like taxonomic or functional composition, phylogenetic structure of the whole community, etc. A dissimilarity matrix composed of pairwise distances between all samples is used for further cluster analysis, classification and study of influence of the experimental factors. In large-scale studies involving tens and hundreds of metagenomes, critical requirements in beta-diversity analysis include high algorithm performance and low memory usage.

For a long time, the standard technological approach for the evaluation of beta-diversity was based on the identification of species in metagenomic samples through 16S rRNA gene sequencing. However, this method has inherent disadvantages including incompleteness of reference databases, presence of multiple copies of 16S rRNA gene in the same genome, discrepancy between phylogenetic trees constructed using 16S rRNA and the other genes and lack of information about the other genes and subsequently metabolic potential of the studied community. An alternative, more informative method is whole-genome sequencing (WGS, “shotgun”) generating millions of reads from the total DNA of the genomes of all organisms inhabiting the environment. The identification of the organisms in the short-read WGS metagenome is commonly based on the alignment or *de novo* assembly [7]. The alignment method is a comparison between sequences of obtained reads and sequences of reference genes or genomes, and has significant drawbacks such as high computational costs and incomplete databases. *De novo* assembly is usually a time-consuming task for such complex data as metagenomes that may contain many unknown or highly similar genomes of organisms with widely varying abundance.

With a rapid increase in data output produced by sequencing technologies, efficient methods for genomic analysis based on *k-*mer composition analysis emerged. Such algorithms work with *k-*mers (oligonucleotide sequences of length *k*, also called *l-*tuples or *n-*grams) obtained directly from metagenomic reads, without pre-mapping or assembly.

In comparison with reference-based methods, the main advantages of *k-*mer based approaches are compressed representation of sequences and inclusion of the entire data volume into analysis (unlike alignment, where only the reads successfully mapped to a reference database influence the result). Among these methods, the most simple and effective for exploratory analysis of large data sets is comparison of sequences by calculation of pairwise dissimilarity between them on the basis of *k-*mer spectrum - a normalized vector of frequencies of occurrences of each *k-*mer in the metagenome. The *k-*mer length is a key factor influencing specificity and efficiency of the comparison. For different intervals of *k*, the respective algorithms have been designed that target different specific tasks. For example, for very short *k-*mers (*k*=4−7) only “rough” estimates are possible: sequence quality check [8, 9], taxonomic separation of individual genomes [10–12] or comparison of metagenomic communities with notably different composition [13–16]. For *k*=15−30, the computational costs associated with the processing of the whole spectrum increase significantly. Two approaches can be applied to reduce them. This problem can be solved in two ways. The first is to select a fraction of *k-*mers that describe the studied data in the most complete way (feature extraction) [17]. Another way is to combine multiple *k-*mers into one feature using a certain principle (different approaches of *k-*mer binning) [18, 19]. In the intermediate range (*k*=7−12), it is still computationally feasible to analyze the complete set of *k-*mers, and the *k-*mer length remains sufficiently specific [12, 20] for comparing distinct genomes.

Among the metagenomic *k-*mer methods, the most simple and effective for exploratory analysis of large data sets is comparison of sequences by calculation of pairwise distances between them on the basis of *k-*mer spectra [21, 22]. In this area, researchers are actively designing fast algorithms for calculating and assessing the *k-*mer spectrum [23, 24]. Most studies are focused on examining clustering of the samples by one or more factors (geography, nutrition, clinical status, etc.) [17, 21].

Since the prevalent approaches for assessment of beta-diversity today are reference-based, an important question is how their results correlate with the *k-*mer methods. In this study, we compared common reference-based methods (based on taxonomic and gene composition, including phylogeny-aware methods) with the *k-*mer approach. We explored how various characteristics of the data influence the results of *k-*mer spectra analysis and identified the advantages of *k-*mer analysis comparing with the reference-based approaches. To evaluate the applicability of *k-*mer-based dissimilarity, metagenome of human gut microbiota was selected, the study of which has great biomedical importance and perspective. Although nowadays intestinal microbiota is one of the most studied among complex microbial communities, many of its components are still not fully identified (among them are uncultured bacteria, phages, fungi and protozoa) [25]. The application of our method revealed significant presence of one of such components - phage - that went undetected by reference-based methods.

## Methods

### Simulated metagenomes

Two set of “shotgun” gut metagenomes were simulated using MetaSim [26]. The high-diversity set included 100 metagenomes generated from the genomes of ten distantly related major bacterial species accounting for more than 90 % of all reads in Chinese group: *Akkermansia muciniphila* ATCC BAA-835, *Alistipes shahii* WAL 8301, *Bacteroides vulgatus* ATCC 8482, *Bifidobacterium adolescentis* ATCC 15703, *Coprococcus sp.* ART55/1, *Eubacterium eligens* ATCC 27750, *Faecalibacterium prausnitzii* L2-6, *Lachnospiraceae bacterium* 1 4 56FAA, *Prevotella copri* DSM 18205 and *Ruminococcus sp.* 18P13.

The simulation included the following steps. First, for each genome, mean and standard deviation of its relative abundance were estimated from the taxonomic composition of the Chinese metagenomes. For each metagenome, ten abundance values were randomly generated under normal distribution with these parameters and the obtained values were normalized to 1 million reads; a total of 100 genera abundance vectors were obtained (see Additional file 1: Table S1). The metagenomes were generated by mixing ten bacterial genomes at the obtained abundance levels and sampling short reads from the genomes using MetaSim with read length 100 bp. Also we performed sampling of these reads with errors (1 % - probability of error in each base).

The low-diversity simulated group included 100 metagenomes generated in a similar way from the genomes of ten closely related major bacterial species accounting for more than 90 % of all reads in the HMP group: *Bacteroides vulgatus* ATCC 8482, *Bacteroides dorei* 5 1 36/D4, *Bacteroides uniformis* ATCC 8492, *Bacteroides stercoris* ATCC 43183, *Bacteroides caccae* ATCC 43185, *Bacteroides ovatus* (strains SD CMC, ATCC 8483 and 3 8 47FAA), *Bacteroides xylanisolvens* XB1A and *Bacteroides thetaiotaomicron* VPI-5482. Bacterial proportions for these simulations are listed in Additional file 1: Table S2.

For single nucleotide polymorphism (SNP) simulations, the same ten reference genomes and abundance values as in the high-diversity dataset were used. Two different models of SNPs introduction were used: “independent” and “phylogenetic”.

In the “independent” SNP model, 64 “mutated” genomes were generated for each reference species by changing nucleotide letter at random positions independently with 0.5 % substitution rate. Thus, the average amount of SNPs between any two of the “mutated” genomes was ∼ 1 %.

In “phylogenetic” SNP model, the procedure was performed in iterations for each reference genome:

- Initialize with a single genome; iteration number = 1.
- Make a copy of each of the genomes available at the step.
- Introduce SNPs to all genomes at random positions.
- Increment iteration number.
- If the iteration number is greater than 6, stop; else return to step b.

After the 6 iterations, 2^6^=64 “mutated” genomes are obtained.

In each model, the random “mutated” genomes of corresponding bacteria were used to generate metagenomes the same way as for high-diversity simulation above.

### Real metagenomic datasets

Two “shotgun” gut metagenomic datasets were analyzed: 129 metagenomes of healthy USA population [27] (referred to as HMP, Illumina platform, read length 101 bp) and 152 metagenomes of Chinese population [28] including healthy and type 2 diabetes individuals (referred to as China, Illumina platform, read length 90 bp). For each sample, the reads were filtered by quality using FASTQ Quality Filter script from FASTX-Toolkit [29] (threshold *QV*≥30 for each nucleotide in a read). For each metagenome, 1 million of high-quality reads was sampled using random _records script from [30]. Comparison of various sampling sizes showed that the selected size of subsampling does not significantly affect the results of the measures’ comparison (see Additional file 2: Figure S5).

### Calculation of *k-*mer vectors and dissimilarity measures

For each metagenome, *k-*mer spectrum was calculated using an *ad hoc* Java program that processes FASTA files read-wise by obtaining *k-*mer counts for each read and adding the counts to a global array (the value of *k* is limited to 15 due to memory consumption). After processing all reads, the counts for reverse-complementary *k-*mers were summed and normalized to a sum of 1. The length of the final feature vector (spectrum) did not exceed *n*=2^2*k*−1^ for odd *k* and *n*=2^2*k*−1^+2^*k*−1^ for even *k* because of reverse-complement *k-*mers.

The obtained *k-*mer spectra were used to calculate pairwise dissimilarity via Bray-Curtis measure defined as:

(1) $$\begin{array}{@{}rcl@{}} BC(x,y)=1-\frac{2\sum\limits_{i=1}^{4^{k}}\min(m_{i}(x),m_{i}(y))}{\sum\limits_{i=1}^{4^{k}}(m_{i}(x)+m_{i}(y))} \end{array} $$

where m is the vector of *k-*mer frequencies normalized to a sum of 1 per metagenome and x, y are two different metagenomes. BC = 0, if the frequencies are equal for all *k-*mers between the metagenomes, and BC = 1, if no common *k-*mers are present in the metagenomes.

### Beta-diversity analysis using reference-based methods

Taxonomic profiling via mapping of metagenomes to a reference genome catalog and coverage analysis was performed as described previously [31], with the only difference: a non-redundant set of 353 genomes of gut microbes was used (Additional file 1: Table S3). The final feature vector for each metagenome included relative abundance of microbial species was normalized to a sum of 100 % (Additional file 1: Tables S4 and S5). Dissimilarity was calculated using these vectors both with Bray-Curtis measure and whole-genome adaptation of the weighted UniFrac metric [31]. Functional profiling was performed as described previously [31] to yield COG (Clusters of Orthologous Groups) [32] relative abundance vectors subsequently used for dissimilarity analysis using Bray-Curtis measure (Additional file 1: Table S6).

An alternative method of taxonomic profiling employed MetaPhlAn v1.7.7 (parameters: -t rel _ab –tax _lev s(g)); here mapping was performed using Bowtie2 v2.0.2 software [33], up to 3 mismatches per read were allowed (mapping results and statistics are in Additional file 1: Table S7). All reference-based methods were summarized in Table 1.

**Table 1:** Types of reference-based analyses used in the study

Type of reference-based analysis	Method	Beta-diversity measure	Designation
Taxonomic profiling	Mapping to a reference catalog	Bray-Curtis	BC TAX (org), (genus)
	of 353 genomes of intestinal microbiota [31]		
		Whole-genome version of	WG UniFrac
		weighted UniFrac	
	Quantitative profiling of unique	Bray-Curtis	BC MetaPhlAn
	clade-specific marker genes (MetaPhlAn) [48]		
Functional profiling	Mapping to Metahit 3,9M catalog of genes [49]	Bray-Curtis	BC COG
	grouped by COGs		

### CrAssphage abundance analysis

All crAssphage genes (GenBank: JQ995537.1) [34] were aligned to the reference gene catalog (similarity criterion: *e*−*value*<1*E*−5, percent of identity <80 *%*, alignment length/query length >0.8, alignment length/subject length >0.8). For each gene, its relative abundance was estimated as a ratio of the total length of the reads mapped to this gene to the total length of the reads mapped to the reference gene catalog. Phage relative abundance was determined as a sum of the relative abundance values of its genes (Additional file 1: Table S8). As an additional method of metagenomic classification, DIAMOND aligner [35] was used (method: BLASTx against nr database with default parameters) in combination with MEGAN classifier [36].

### Statistical analysis

Statistical analysis was implemented in R. The code is available at:

http://download.ripcm.com/Dubinkina_2015_suppl_data/ and https://github.com/Zireae1/kmer_project/.

### Ethical approval

The sampling procedure was approved by the Ethical Committee for Clinical Research from the Peking University Shenzhen Hospital, Shenzhen Second People’s Hospital and Medical Research Center of Guangdong General Hospital (from the [28] reference), and Enrollments were approved by the Institutional Review Boards of the two recruitment centers (Baylor College of Medicine, Houston, TX and Washington University, St. Louis, MO) (from [27]).

## Results

To compare the *k-*mer based metagenomic beta-diversity measure with traditional reference-based methods we conducted a series of computational experiments on simulated and real data. To validate the *k-*mer measure and find an optimal value of *k*, we simulated metagenomes from prevalent human gut bacterial genomes. Then the method was applied for the analysis of a group of real human gut metagenomes sequenced in two large-scale projects: China population (n = 152) [28] and HMP (n = 129) [27].

### Comparison of beta-diversity measures for simulated metagenomes

There is considerable variation between *k-*mer spectra for genomes of distinct bacterial species due to the differences in the gene content, amino acid coding preferences, etc. [37]. Supposedly, in the case of a metagenome including sufficiently covered genomes of multiple species, one should observe significant accordance between *k-*mer spectrum of the metagenome and its taxonomic composition. To verify this hypothesis, we simulated several datasets with different degrees of community richness and applied the Bray-Curtis measure (a common microbial ecological index) for both taxonomic and *k-*mer profiles to compare the two respective dissimilarity matrices (see Methods for details).

#### Simulation 1: high-diversity communities

The first synthetic dataset included 100 metagenomes generated by randomly sampling “reads” from ten genomes of common phylogenetically distant human gut bacteria (see Methods for details). Comparison of the two approaches showed that, as *k* increases, so does the correlation value between the two dissimilarity matrices based on *k-*mers and taxonomic composition. With high values of *k*, the two matrices are highly similar (e.g. for *k*=10, Mantel test: Spearman correlation *r*=0.88,*p*=0.001, see Additional file 3: Figure S1 and Additional file 4: Figure S2).

#### Simulation 2: sequencing errors and SNPs

Besides the considerable number of species within microbiota, the other factors contributing to the diversity of metagenomic *k-*mers are presence of mutations and sequencing errors in reads. Therefore, we conducted two experiments by introducing artificial SNPs into genomes and, separately, random single-nucleotide changes (“sequencing errors”) into the reads in order to explore their influence on the correlation of beta-diversity estimates using *k-*mer and taxonomic methods. Datasets from simulation 1 were used here.

For sequencing errors modeling, the reads for each metagenome were simulated with per-nucleotide substitution rate of 1 % (a typical order of value for most modern DNA sequencing platforms [38]). Introduction of such “errors” did not lead to a significant change in correlation between the two methods (from 0.88 to 0.87, for *k*=10).

For SNP modeling, bacterial genomes with 1 % of randomly introduced single-nucleotide substitutions (according to an estimate for gut bacteria [39]) were used to generate simulated metagenomes with the same abundance proportions as in simulation 1. We employed two different models of SNPs introduction - “independent” and “phylogenetic”. With the former simulation being more straight-forward, “phylogenetic” approach was developed to model the accumulation of mutations in bacterial species during evolutionary process (see Methods for details). The results of simulations showed that, independent of the model choice, in general SNPs had a minor effect on *k-*mer spectra comparable to the effect of simulated sequencing errors: correlation between the *k-*mer and taxonomic methods decreased from 0.931 to 0.929 for “independent” model and 0.927 for “phylogenetic” model (Additional file 3: Figure S1A,B). Noteworthy, introduction of SNPs had a more pronounced effect on metagenomes with highly similar taxonomic composition. This was particularly marked when the SNP rate was increased to 10 % (Additional file 3: Figure S1C).

#### Simulation 3: low-diversity communities

The second synthetic dataset included 100 metagenomes generated by randomly sampling “reads” from ten genomes of common phylogenetically close human gut bacteria - belonging to the same genus - *Bacteroides* (see Methods for details). The correlation between the methods was found to be lower for such homogeneous community than for a heterogeneous one (for *k* = 10, Mantel test: Spearman correlation *r*=0.82,*p*=0.001). The correlation value tends to increase with *k* but does not achieve the level of simulation 1 (for *k*=10,*r*=0.88, see Additional file 3: Figure S1). It suggests that higher values of *k* should be used to increase accuracy; however, the size of the feature vector increases as 4^*k*^, hence the computational time quickly becomes unacceptable. To select the optimal *k* value, we evaluated the correlation between *k-*mer and taxonomic dissimilarity matrices together with the computational time of *k-*mer matrix generation for *k*=5−12 using both high- and low-diversity simulated datasets (see Additional file 5: Figure S3). As the results in both simulations showed, with *k* = 11 the dissimilarity matrices are highly correlated while the computational time is still acceptable (on a single computation core, the calculation of *k-*mers spectra for one sample took about ten seconds (for comparison the Jellyfish counter [23] with parameters: -m 11 -s 10000 -t 32 (hash size was optimized for the value of *k*) took about 80 seconds to calculate the spectra) Further statistical analysis - calculation of dissimilarity matrix - took about 1 - 10 minutes, see Additional file 5: Figure S3). At the same time, it is the highest value practically acceptable in terms of memory usage: for *k*=11, the spectra occupied ∼4 Gb of memory in R environment, but for *k* = 12 - as much as 15 Gb. Considering these observations we selected *k*=11 for further analyses.

### Comparison of beta-diversity measures on real human gut metagenomes

After testing the method on simulated data, we examined two real human gut datasets from large-scale metagenomic projects: China [28] and HMP [27], with the former cohort representing more diverse microbial community structures than the latter [31]. Using this data, the pairwise dissimilarity matrix obtained by the *k-*mer approach with Bray-Curtis measure (refered as **BC kmer** in the Figures and further in the text) was compared with the dissimilarity matrices obtained by each of the four methods based on taxonomic and functional reference (see Table 1).

**Fig. 1:**
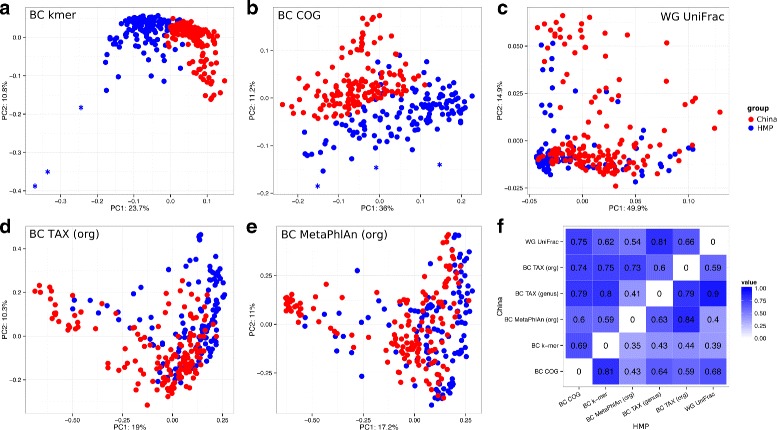
Variation of metagenomes using different dissimilarity measures. PCoA plots for different dissimilarity measures: **a** BC kmer, **b** BC COG, **c** WG UniFrac, **d** BC TAX (org), **e** BC MetaPhlAn (org). Three samples-outliers are marked with asterisks. **f** Heatmap of Spearman correlation coefficient between dissimilarity matrices obtained using different measures (the upper triangle of matrix represents coefficients for China, the lower - for HMP)

**Fig. 2:**
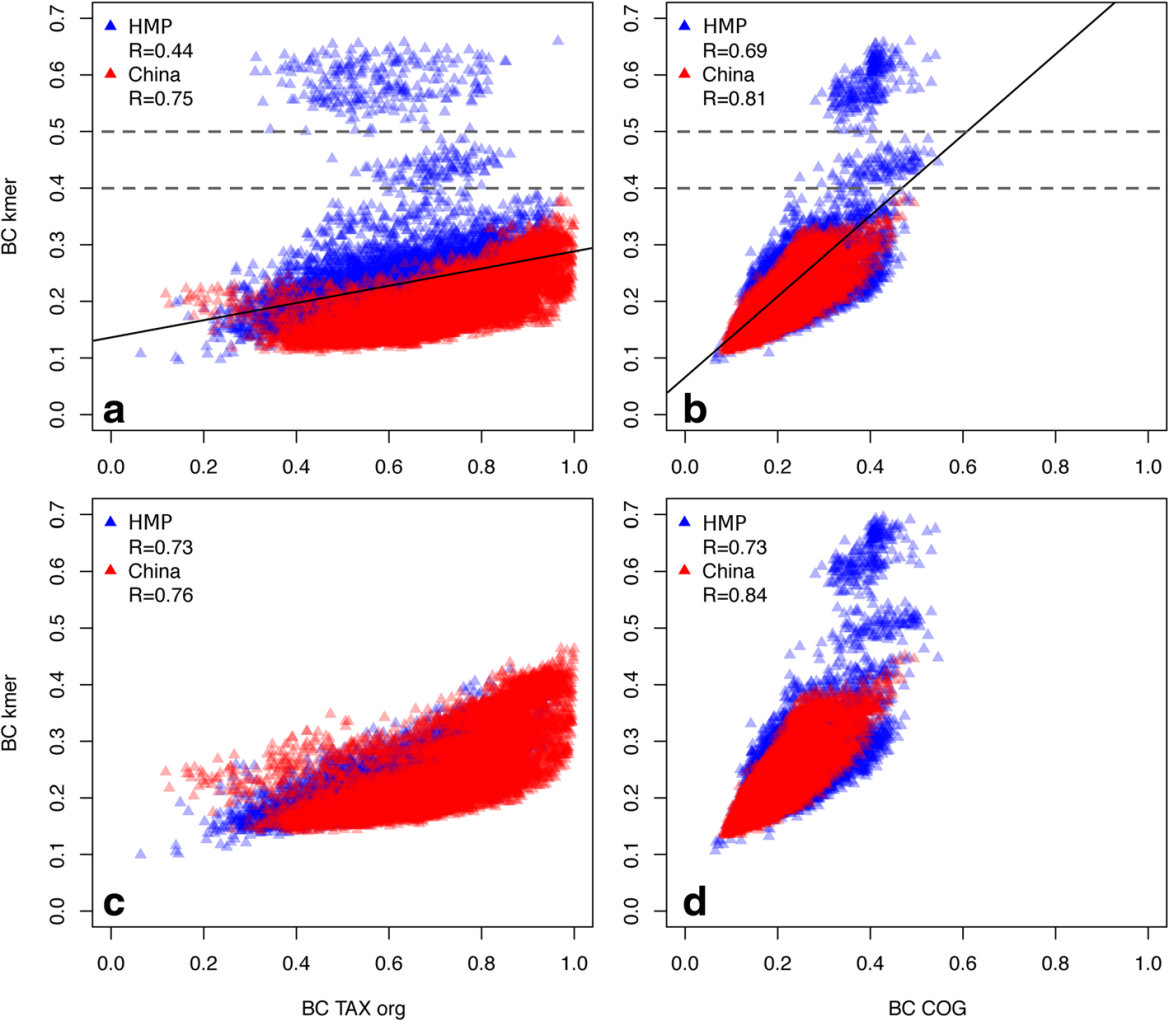
Comparison of pairwise difference measures obtained by *k-*mer and reference-based methods. For each plot, Y-axis represents *k-*mer distance, X-axis - distance by one of the reference-based methods. Distribution of dissimilarity measures is shown for **a** BC kmer for all reads and BC TAX (org); **b** BC kmer for all reads and BC COG; **c** BC kmer for reads mapped to the catalog of genomes and BC TAX (org); **d** BC kmer for reads mapped to the catalog of genes and BC COG

To visualize the distributions of beta-diversity values, we applied two types of scatter plots. The first type is a basic principle coordinate analysis (PCoA) plot constructed using a single dissimilarity measure, with dots representing distinct metagenomes (e.g. Fig. 1a). On the second type of plot, two dissimilarity measures are compared: each triangle corresponds to a pair of metagenomes, one measure is plotted against the other (Fig. 2a, Additional file 6: Figure S4). Samples from the two studies (China and HMP) tended to cluster separately by functional, as well as by *k-*mer composition, but not by taxonomic composition (Fig. 1a, b, c, d). Therefore, the two cohorts were further analyzed separately. Another interesting fact was that 3 of the outliers (all from HMP group) present on *k-*mer scatter plot were also on the periphery of COG scatter plot but not of the taxonomic scatter plot (Fig. 1a, b; outliers marked with asterisks). These samples were examined in details.

Comparison of the five beta-diversity measures showed that the *k-*mer measure has a significant similarity with each of the reference-based ones (Mantel test, Spearman correlation, *p*<0.01, Fig. 1f). The closest was the measure based on COG composition. For the Chinese group, the correlation values tended to be higher than for the HMP group in all comparisons (Fig. 1f). The phylogeny-aware metric WG UniFrac was among the most dissimilar (*r*=0.39 for HMP, *r*=0.62 for China).

#### Influence of reads mappability

To assess the contribution of the unmapped reads to the results, *k-*mer spectra were also computed only using the reads that successfully mapped to the corresponding catalog (fraction of mapped reads: for HMP group - 49 ± 17 % for genome catalog, 60 ± 5 % for gene catalog, for China - 49 ± 12 %, 61 ± 6 %, respectively; values are given in median ± s.d. here). This analysis led to interesting results (Fig. 2). First, we observed an equalization of BC TAX org vs. BC kmer correlation between the two cohorts (0.74 for HMP and 0.77 for China). Therefore, fraction of unmapped reads appears to be one of the major factors contributing to the difference between the cohorts. This parameter is dependent on the representability of the reference catalog and quality of sequencing run.

Second, we assessed the shift of each outlier in the direction of the central cloud of points. Quantitatively, for each outlier we calculated the BC kmer difference value: the difference between its BC kmer value and the linearly interpolated middle of the cloud obtained for the same reference-based value (Fig. 2). For comparison with BC TAX, the BC kmer difference decreased significantly - from 0.31 ± 0.09 to 0.03 ± 0.04 (Wilcoxon test, *p*=2.2*E*−16). For comparison with BC COG, the BC kmer difference changed slightly: from 0.34 ± 0.08 to 0.39 ± 0.07. Correspondingly, a group of pairs-outliers mentioned above moved into the central cloud of points in the BC TAX org vs. BC kmer comparison, but did not change their visual location in BC COG vs. BC kmer comparison.

This observation is in agreement with the fact that the gene reference catalog is more complete than the genome reference catalog and the percentage of mapping to the gene catalog is higher (49 ± 17 % vs 60 ± 5 % for HMP and 49 ± 12 % vs. 61 ± 6 % for China, respectively, Wilcoxon test, *p*=2.2*E*−16). Presumably, the presence of pairs-outliers could be caused by *k-*mers from certain dominant sequences that are present in the reference base of genes but not genomes. We investigated these outliers in details.

#### Investigation of samples-outliers

The total human gut metagenome is a phylogenetically diverse structure including not only the sequences of bacterial genomes but also ones from bacterial mobilome (phages, plasmids, etc.), fungi, protozoa, traces of DNA of dietary origin, host. Our reference genome catalog partly accounts for such non-microbial components by including the genomes of several common intestinal eukaryotes - clinically relevant yeasts *Candida* (3 genomes) and protozoan *Blastocystis* (1 genome; see Methods for details). However, many sequences are not present in our genome catalog, particularly viral genomes. Therefore, in our analysis the potential reads of viral origin would not contribute to the taxonomic difference but would change the *k-*mer spectrum. Recently, a new bacteriophage was discovered - crAssphage - shown to be a sole major dominant of the human gut viriome [34]. Moreover, its presence was estimated for the HMP metagenomes analyzed in our work: crAssphage genome amounts for up to 20 % of the reads for this group. Obviously, such a prevalent genome should have a significant influence on *k-*mer spectra and thus on our comparison of the beta-diversity measures.

**Fig. 3:**
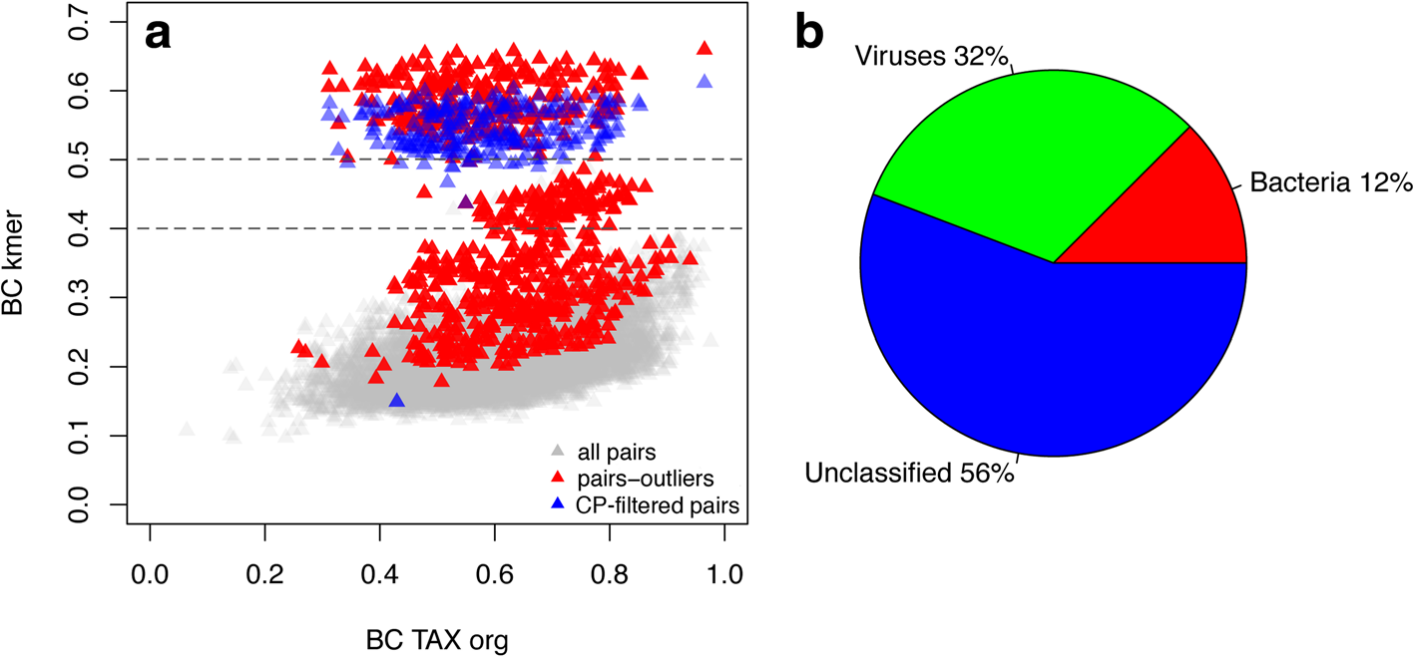
Analysis of samples-outliers. **a** Distribution of pairwise dissimilarity obtained using *k-*mer and taxonomic composition for HMP cohort. Different colors indicate groups of dissimilarities for: all HMP pairs, pairs-outliers - where at least one of the samples belonged to the phage-enriched group; CP-filtered pairs - extreme outliers (all pairs with *k-*mer distance > 0.5) after removal of *k-*mers from reads mapped to crAssphage (CP) genome; **b** Composition of sample SRS062427 according to the combined results from two analyses (mapping to genome catalog and DIAMOND + MEGAN)

Basing on the available data on the abundance of crAssphage in HMP samples (see Methods for details), the cohort was split into two groups - with high phage abundance (*n*=5,5−20 *%* of crAssphage reads) and with low phage abundance (*n*=124,<5 *%* of crAssphage reads). The whole group of extreme outliers was found to consist of the pairs where at least one of the samples belonged to the phage-enriched group (Fig. 3a, chi-square test: *X*^2^=802.97,*p*=2.2*E*−16). Moreover, the outliers on Fig. 1a were also found to be the samples with high fraction of crAssphage reads (see Additional file 1: Table S8).

As the extreme outliers were found to be generated by pairs including at least one of the two samples (SRS062427 and SRS014287, see Fig. 1a), these samples were analyzed in detail. The reads that did not map to the genome catalog (86 % and 88 % from the total read number, respectively) were subject to metagenomic classification using an alternative method - using DIAMOND alignment and MEGAN classifier algorithms (see Methods). As a result, additionally 35 % and 29 % of the reads were identified as crAssphage (Fig. 3b). To further confirm the contribution of high phage fraction to formation of outliers, we subtracted the *k-*mers of the crAssphage reads from *k-*mer spectra of the samples. Indeed, such operation significantly decreased the *k-*mer-based dissimilarity for the respective pairs (0.57 ± 0.08 to 0.53 ± 0.07, one-tailed Wilcoxon test, *p*<2.2*E*−16, Fig. 3a).

## Discussion

Here we have developed an algorithm for assessing pairwise dissimilarity of “shotgun” metagenomes basing on *k-*mer spectrum and compared it with commonly used reference-based approaches. The comparison was performed using various measures (Bray-Curtis dissimilarity and whole-genome adaptation of UniFrac) on a set of simulated metagenomes as well as on real metagenomes from two large-scale human gut microbiota studies.

For simulated metagenomes, we showed that *k* = 11 is an optimal value in terms of balancing between the resolution of the method and computational time. This value of *k* performed well for both high- and low-diversity simulated metagenomes; however, for low-diversity simulations the dissimilarity matrices based on *k-*mer method and taxonomic composition were less correlated (Spearman correlation *r*=0.94 and *r* = 0.87 for high- and low-diversity, respectively). This fact was likely due to the decreased diversity of *k-*mers and thus reduced differentiating resolution. For real gut metagenomes with complex community structure, the *k-*mer approach allows to delineate the samples with a wide range of functional composition, as demonstrated on two international cohorts (HMP and Chinese population). On the other hand, we observed that *k-*mers are less correlated with taxonomic composition than with functional (gene-based) one. We speculate that this difference could be associated with significant subspecies-level genomic diversity of gut microbes: a recent analysis of publicly available metagenomic data showed that the average gene variation between individuals across 11 abundant species was as high as 13 ± 4.5 % [40]. The *k-*mer frequencies as well as the gene relative abundance features are sensitive to gene content variation, while in the case of species relative abundance features it would be ignored.

Besides gene presence/absence, another common form of genomic variability is SNPs. We attempted to model their influence on *k-*mer beta-diversity. Theoretically, introduction of SNPs would lead to change in frequencies of *k-*mers and thus deteriorate correlation between *k-*mer and reference-based dissimilarity. In our simulations, when 1 % SNPs were introduced to simulated datasets (according to an estimate for gut bacteria [39]), the correlation between the methods dropped slightly (from *r*=0.938 to *r*=0.935), independent of whether the evolutionary character of SNP accumulation was considered during modeling or not. However, for real metagenomes the correlation between the methods was lower (HMP: *r*=0.73, China: *r*=0.76). These results suggest the existence of other real-life effects having stronger influence on the correlation than SNPs (not only other types of genetic polymorphisms like indels but also including technical factors, etc.).

A major advantage of the *k-*mer approach is that it exploits the totality of the reads - unlike reference-based methods that inherently discard the reads that failed to map to the reference catalog - and thus the information contained within them. Such a feature promotes the application of the *k-*mer approach as a tool for assessing the representability of the reference set for given metagenomes. Currently representative sets of reference genomes are available for microbial communities of few environments (e.g. human gut). However, recent discoveries imply that the so called reference genomes do not capture a wide intra-species level variation even for this extensively examined community [41, 42]. The situation is even worse for less popular environments - like marine ecosystems [43] or human skin [44]: reference catalogs for their microbiota are considerably less complete, thus rendering beta-diversity assessment difficult.

We propose to assess the representability of a reference genome catalog via examining the *k-*mer content of the metagenomic reads mapped to it. On the analyzed gut metagenomic data, we observed that *k-*mer spectra of the mapped reads produced dissimilarity profiles that had higher correlation with those obtained with taxonomic composition than the *k-*mer spectra of the whole set of reads. However, lower correlation between the two methods observed for some pairs of samples suggested the presence of dominant genomic sequences not included in the reference catalog. Detailed analysis showed that these outliers corresponded to the HMP samples enriched in crAssphage, a recently discovered gut bacteriophage [34]; the genome of this phage was not included in the respective reference catalog.

Subtraction of the crAssphage *k-*mers moved the outliers towards the main cloud of points but not into it completely (BC kmer difference decreased by 90 ± 10 %). Presumably, such incomplete compensation can be linked to high level of genomic variability inherent to gut phages [45]: originally the consensus crAssphage genome was obtained by combined assembly of 12 metagenomes from individuals not included in our groups [46], so its sequence in the latter might be quite distant than all the crAssphage-related *k-*mers in our analysis. Additionally, over 50 % of the reads remain unidentified by two different methods (mapping to reference genome catalog and DIAMOND+MEGAN-based pipeline) they can correspond to genome(s) contributing to formation of outliers.

Considering the gene catalog, dedicated analysis of the reads mapped to reference genes did not lead to shift of outliers (BC kmer difference slightly increased by 16 ± 9 %). First, a likely reason for this is that the crAssphage sequences were included in the catalog: search for crAssphage genes in the reference gene catalog (see Methods) identified highly similar hits for 70 of the 80 phage genes (182 catalog genes in total) that were detected in at least one metagenome. Second, the gut microbial gene catalog was originally constructed from the contigs assembled from total DNA reads [49] and is known to contain not only the bacterial genes, but viral and eukaryotic, too.

Interestingly, our results also imply that the Chinese cohort lacks metagenomes with such high prevalence of this phage, provoking speculations on world-wide phage phylogeography. While no clinical associations for crAssphage have been described to date, omission of phage components could be a significant miss in biomedical studies of microbiota. There is a growing understanding that gut phages play an important role in the ecology of “phage-gut microbiota-human” system and include potential biomarkers; they are able to transfer clinically important bacterial genes - e.g. antibiotic resistance and pathogenicity determinants [47]. Application of our reference-free *k-*mer approach can facilitate early detection of such sequences in biomedical diagnostics data and discovery of novel biomarkers.

Our approach is not only applicable to metagenomes from an arbitrary environment, but is indispensable for dissimilarity and cluster analysis of communities with poorly described components. The approach allows to detect a major presence of an unknown organism and/or virus in a metagenome. We suggest that the approach should be introduced as a necessary method of “shotgun” metagenome composition analysis complementary to reference mapping in order to avoid biases associated with unrepresentative reference database.

Although we did not find evidence for outliers caused by technical issues in the examined datasets, the approach can also be used for primary detection of metagenomes with abnormal composition caused by high abundance of host DNA (e.g. in case of inflammatory process or specific to biopsy material), DNA of dietary origin (undigested food) and technical artifacts (e.g. dominance of sequencing adapters).

Finally, comparison of the metagenomes basing on *k-*mer spectrum provides more information than mapping to reference sequence catalogs. Essentially, *k-*mer analysis is a feature extraction procedure applied to metagenomic reads. The produced set of features (*k-*mer spectrum) is several orders of magnitude larger than one yielded in reference-based approaches. Therefore, it provides higher discriminative resolution that opens a promising opportunity for developing a new generation of methods for metagenomic analysis, and our method makes a step towards understanding of how to explore such high-dimensional feature space efficiently.

## Conclusions

Analysis of *k-*mer spectra for both simulated and real “shotgun” metagenomes showed that this method allows quick assessment of the pairwise dissimilarity of such datasets. Simulations show that the method is robust to variability introduced by sequencing errors and genomic mutations. The obtained dissimilarity matrix can be used not only for cluster analysis and classification purposes, but also for early detection of major unknown components and quality control of reference-based approaches. It is recommended that the method should be included as a complementary step in high-throughput computational pipelines for metagenomic data analysis.

## Additional files

Additional file 1
**Supplementary tables.** Supplementary **Table S1.** Bacterial abundances in Simulation 1 (high-diversity communities). Supplementary **Table S2.** Bacterial abundances in Simulation 2 (low-diversity communities). Supplementary **Table S3.** List of genomes in taxonomic catalog for human gut. Supplementary **Table S4.** Taxonomic composition for real dataset (organism level). Supplementary **Table S5.** Taxonomic composition for real dataset (genus level). Supplementary **Table S6.** Functional composition for real dataset (COG). Supplementary **Table S7.** Taxonomic composition for real dataset by MetaPhlAn (organism level). Supplementary **Table S8.** Mapped read counts and percentage of mapping on taxonomic and functional catalog and phage genome. (XLS 2693 kb)

Additional file 2
**Figure S5.** Correlation between *k-*mer-based dissimilarity matrix obtained using the original full metagenomic readsets and their subsampled versions. The same amount of reads was repeatedly sampled from 20 randomly selected metagenomes (total amount ranging from 30 to 50 mln reads). (PNG 69 kb)

Additional file 3
**Figure S1.** Comparison of pairwise difference measures obtained by *k-*mer and taxonomic composition (50 simulated metagenomes). For each pair Y-axis represents *k-*mer dissimilarity, X-axis represents dissimilarity by taxonomic composition. *k*=10 A) using the original bacterial genomes; B) using the bacterial genomes with 1 % of SNPs. (PNG 451 kb)

Additional file 4
**Figure S2.** Comparison of pairwise dissimilarity measures obtained by *k-*mer and taxonomic composition for simulated for high- and low-diversity metagenomes. As seen, satisfactory correlation of *k-*mers with taxonomic composition can be obtained only at relatively high values of *k*. (PNG 233 kb)

Additional file 5
**Figure S3.** Correlation between *k-*mer and taxonomic composition dissimilarity matrices, as well as *k-*mer dissimilarity matrix computation time with varying values of *k*. All computations were performed on a compute node with CPU Opteron 6176 2.3 GHz (24 cores) and 64 Gb RAM. (PNG 185 kb)

Additional file 6
**Figure S4.** Comparison of dissimilarity measures obtained by *k-*mer and 3 reference-based methods: BC MetaPhlAn genus, BC MetaPhlAn org and WG UniFrac. For each plot, Y-axis represents *k-*mer dissimilarity, X-axis - dissimilarity using one of reference-based methods. (PNG 925 kb)

## Abbreviations

16S rRNA: 16S ribosomal ribonucleic acid
BC: Bray-Curtis dissimilarity measure
BC kmer: BC based on *k-*mer spectrum
BC TAX: BC based on taxonomic composition, obtained by mapping to catalog of genomes
BC MetaPhlAn: BC based on taxonomic composition, obtained by mapping to clade-specific marker genes
BC COG: BC based on functional composition, obtained by mapping to catalog of COG (Clusters of Orthologous Groups) genes
DNA: Deoxyribonucleic acid
SNP: Single Nucleotide Polymorphism
WGS: whole genome sequencing, “shotgun”
WG UniFrac: whole genome adaptation of UniFrac measure

## References

[1] Dick GJ, Andersson AF, Baker BJ, Simmons SL, Thomas BC, Yelton AP (2009). Community-wide analysis of microbial genome sequence signatures. Genome Biol.

[2] Park EJ, Kim KH, Abell GCJ, Kim MS, Roh SW, Bae JW (2010). Metagenomic Analysis of the Viral Communities in Fermented Foods. Appl Environ Microbiol.

[3] Singh B, Gautam SK, Verma V, Kumar M, Singh B (2008). Metagenomics in animal gastrointestinal ecosystem: Potential biotechnological prospects. Anaerobe.

[4] Morgan XC, Segata N, Huttenhower C (2013). Biodiversity and functional genomics in the human microbiome. Trends in genetics: TIG.

[5] Riesenfeld CS, Schloss PD, Handelsman J (2004). Metagenomics: Genomic Analysis of Microbial Communities. Annu Rev Genet.

[6] Lozupone C, Lladser ME, Knights D, Stombaugh J, Knight R (2011). UniFrac: an effective distance metric for microbial community comparison. ISME J.

[7] Teeling H, Glöckner FO (2012). Current opportunities and challenges in microbial metagenome analysis–a bioinformatic perspective. Brief Bioinform.

[8] Yang B, Peng Y, Leung HC-M, Yiu SM, Chen JC, Chin FY-L (2010). Unsupervised binning of environmental genomic fragments based on an error robust selection of l-mers. BMC Bioinformatics.

[9] Plaza Onate F, Batto JM, Juste C, Fadlallah J, Fougeroux C, Gouas D (2015). Quality control of microbiota metagenomics by k-mer analysis. BMC Genomics.

[10] Zhou F, Olman V, Xu Y (2008). Barcodes for genomes and applications. BMC Bioinformatics.

[11] Pride DT, Meinersmann RJ, Wassenaar TM, Blaser MJ (2003). Evolutionary implications of microbial genome tetranucleotide frequency biases. Genome Res.

[12] Alsop EB, Raymond J (2013). Resolving prokaryotic taxonomy without rRNA: longer oligonucleotide word lengths improve genome and metagenome taxonomic classification. PloS One.

[13] Cui H, Zhang X (2013). Alignment-free supervised classification of metagenomes by recursive SVM. BMC Genomics.

[14] Silva GGZ, Cuevas DA, Dutilh BE, Edwards RA (2014). FOCUS: an alignment-free model to identify organisms in metagenomes using non-negative least squares. PeerJ.

[15] Langenkämper D, Goesmann A, Nattkemper TW (2014). AKE - the Accelerated k-mer Exploration web-tool for rapid taxonomic classification and visualization. BMC Bioinformatics.

[16] Liao R, Zhang R, Guan J, Zhou S (2014). A New Unsupervised Binning Approach for Metagenomic Sequences Based on N-grams and Automatic Feature Weighting. IEEE/ACM Trans Comput Biol Bioinformatics.

[17] Seth S, Välimäki N, Kaski S, Honkela A (2014). Exploration and retrieval of whole-metagenome sequencing samples. Bioinformatics (Oxford, England).

[18] Ames SK, Hysom DA, Gardner SN, Lloyd GS, Gokhale MB, Allen JE (2013). Scalable metagenomic taxonomy classification using a reference genome database. Bioinformatics (Oxford, England).

[19] Wu YW, Ye Y (2011). A novel abundance-based algorithm for binning metagenomic sequences using l-tuples. J Comput Biol J Comput Mol Cell Biol..

[20] Jiang B, Song K, Ren J, Deng M, Sun F, Zhang X (2012). Comparison of metagenomic samples using sequence signatures. BMC Genomics.

[21] Wang Y, Liu L, Chen L, Chen T, Sun F (2014). Comparison of metatranscriptomic samples based on k-tuple frequencies. PloS One.

[22] Vinga S, Almeida J (2003). Alignment-free sequence comparison–a review. Bioinformatics.

[23] Marçais G, Kingsford C (2011). A fast, lock-free approach for efficient parallel counting of occurrences of k-mers. Bioinformatics (Oxford, England).

[24] Audano P, Vannberg F (2014). KAnalyze: a fast versatile pipelined k-mer toolkit. Bioinformatics (Oxford, England).

[25] Bäckhed F, Ley RE, Sonnenburg JL, Peterson DA, Gordon JI (2005). Host-bacterial mutualism in the human intestine. Science (New York, N.Y.).

[26] Richter DC, Ott F, Auch AF, Schmid R, Huson DH (2008). MetaSim: a sequencing simulator for genomics and metagenomics. PloS One.

[27] Structure, function and diversity of the healthy human microbiome. Nature. 2012; 486(7402):207–14. doi:10.1038/nature11234. 10.1038/nature11234 PMC3564958 22699609

[28] Qin J, Li Y, Cai Z, Li S, Zhu J, Zhang F (2012). A metagenome-wide association study of gut microbiota in type 2 diabetes. Nature.

[29] Pearson WR, Wood T, Zhang Z, Miller W (1997). Comparison of DNA sequences with protein sequences. Genomics.

[30] Hansen MA, Oey H, Fernandez-Valverde S, Jung CH, Mattick JS. Biopieces: A Bioinformatics Toolset and Framework. http://www.biopieces.org.

[31] Tyakht AV, Kostryukova ES, Popenko AS, Belenikin MS, Pavlenko AV, Larin AK (2013). Human gut microbiota community structures in urban and rural populations in Russia. Nat Commun.

[32] Tatusov RL (2000). The COG database: a tool for genome-scale analysis of protein functions and evolution. Nucleic Acids Res.

[33] Langmead B, Salzberg SL (2012). Fast gapped-read alignment with Bowtie 2. Nat Methods.

[34] Dutilh BE, Cassman N, McNair K, Sanchez SE, Silva GGZ, Boling L, et al.A highly abundant bacteriophage discovered in the unknown sequences of human faecal metagenomes. Nat Commun. 2014;5. doi:10.1038/ncomms5498. 10.1038/ncomms5498 PMC4111155 25058116

[35] Buchfink B, Xie C, Huson DH (2014). Fast and sensitive protein alignment using DIAMOND. Nat Methods.

[36] Huson DH, Auch AF, Qi J, Schuster SC (2007). MEGAN analysis of metagenomic data. Genome Res.

[37] Chor B, Horn D, Goldman N, Levy Y, Massingham T (2009). Genomic DNA k-mer spectra: models and modalities. Genome Biol.

[38] Scholz MB, Lo CC, Chain PS (2012). Next generation sequencing and bioinformatic bottlenecks: the current state of metagenomic data analysis. Curr Opinion Biotechnol.

[39] Schloissnig S, Arumugam M, Sunagawa S, Mitreva M, Tap J, Zhu A (2013). Genomic variation landscape of the human gut microbiome. Nature.

[40] Zhu A, Sunagawa S, Mende DR, Bork P (2015). Inter-individual differences in the gene content of human gut bacterial species. Genome Biol.

[41] Greenblum S, Carr R, Borenstein E (2015). Extensive Strain-Level Copy-Number Variation across Human Gut Microbiome Species. Cell.

[42] Nielsen HBR, Almeida M, Juncker AS, Rasmussen S, Li J, Sunagawa S (2014). Identification and assembly of genomes and genetic elements in complex metagenomic samples without using reference genomes. Nat Biotechnol.

[43] Sunagawa S, Coelho LP, Chaffron S, Kultima JR, Labadie K, Salazar G (2015). Ocean plankton. Structure and function of the global ocean microbiome. Science (New York, N.Y.).

[44] Leung MHY, Wilkins D, Lee PKH (2015). Insights into the pan-microbiome: skin microbial communities of Chinese individuals differ from other racial groups. Sci Rep.

[45] Minot S, Sinha R, Chen J, Li H, Keilbaugh SA, Wu GD (2011). The human gut virome: inter-individual variation and dynamic response to diet. Genome Res.

[46] Reyes A, Haynes M, Hanson N, Angly FE, Heath AC, Rohwer F (2010). Viruses in the faecal microbiota of monozygotic twins and their mothers. Nature.

[47] Modi SR, Lee HH, Spina CS, Collins JJ (2013). Antibiotic treatment expands the resistance reservoir and ecological network of the phage metagenome. Nature.

[48] Segata N, Waldron L, Ballarini A, Narasimhan V, Jousson O, Huttenhower C (2012). Metagenomic microbial community profiling using unique clade-specific marker genes. Nat Methods.

[49] Qin J, Li R, Raes J, Arumugam M, Burgdorf KS, Manichanh C (2010). A human gut microbial gene catalogue established by metagenomic sequencing : Article : Nature. Nature.

