# Supplementary figures and images for: Assessment of k-mer spectrum applicability for metagenomic dissimilarity analysis
Source: BMC Bioinformatics. 2016 Jan 16;17:38. doi: 10.1186/s12859-015-0875-7 (PMC4715287; doi:10.1186/s12859-015-0875-7)

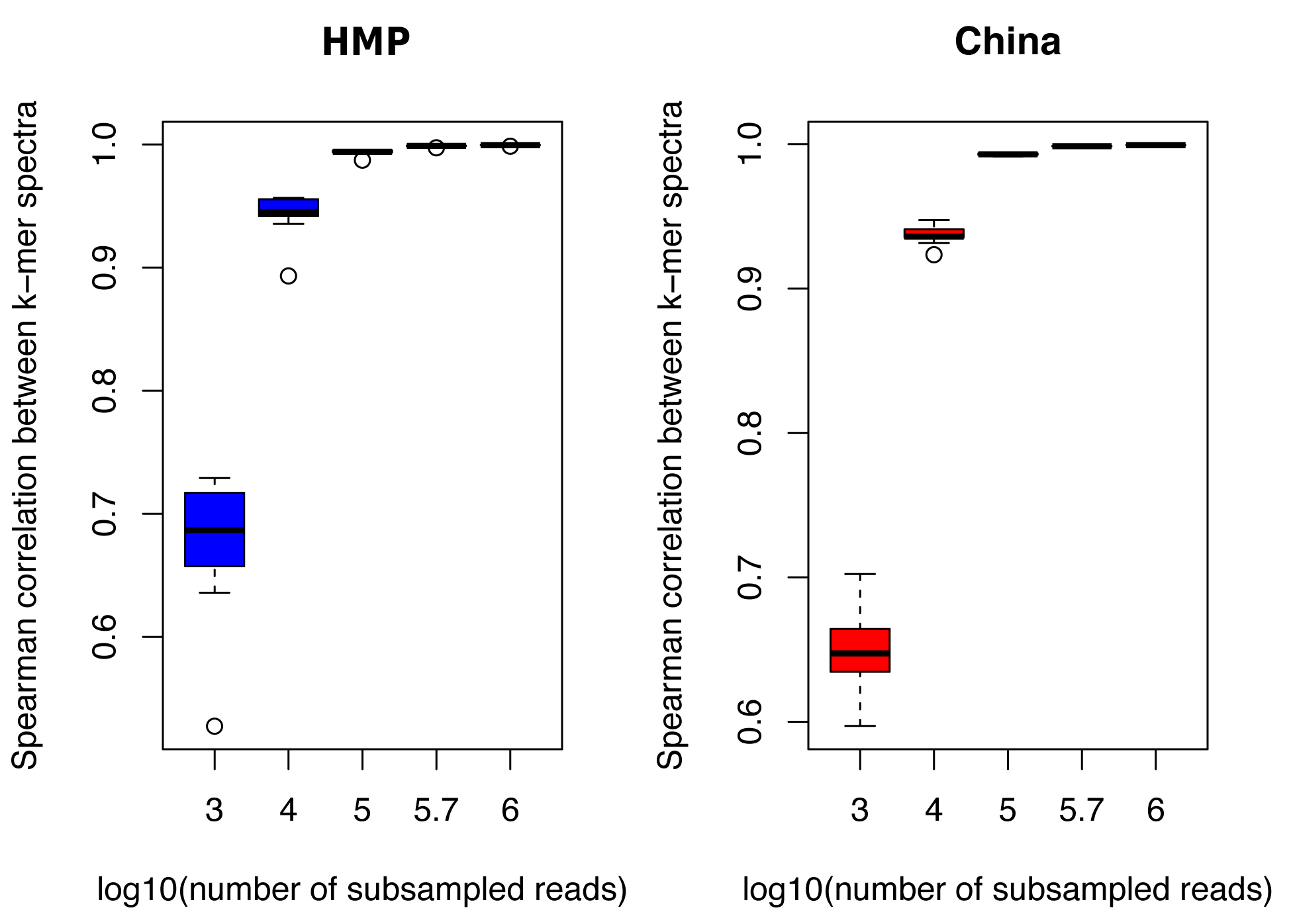

Supplement: Additional file 2 — Figure S5. Correlation between k-mer-based dissimilarity matrix obtained using the original full metagenomic readsets and their subsampled versions. The same amount of reads was repeatedly sampled from 20 randomly selected metagenomes (total amount ranging from 30 to 50 mln reads). (PNG 69 kb) [file 12859_2015_875_MOESM2_ESM.png]

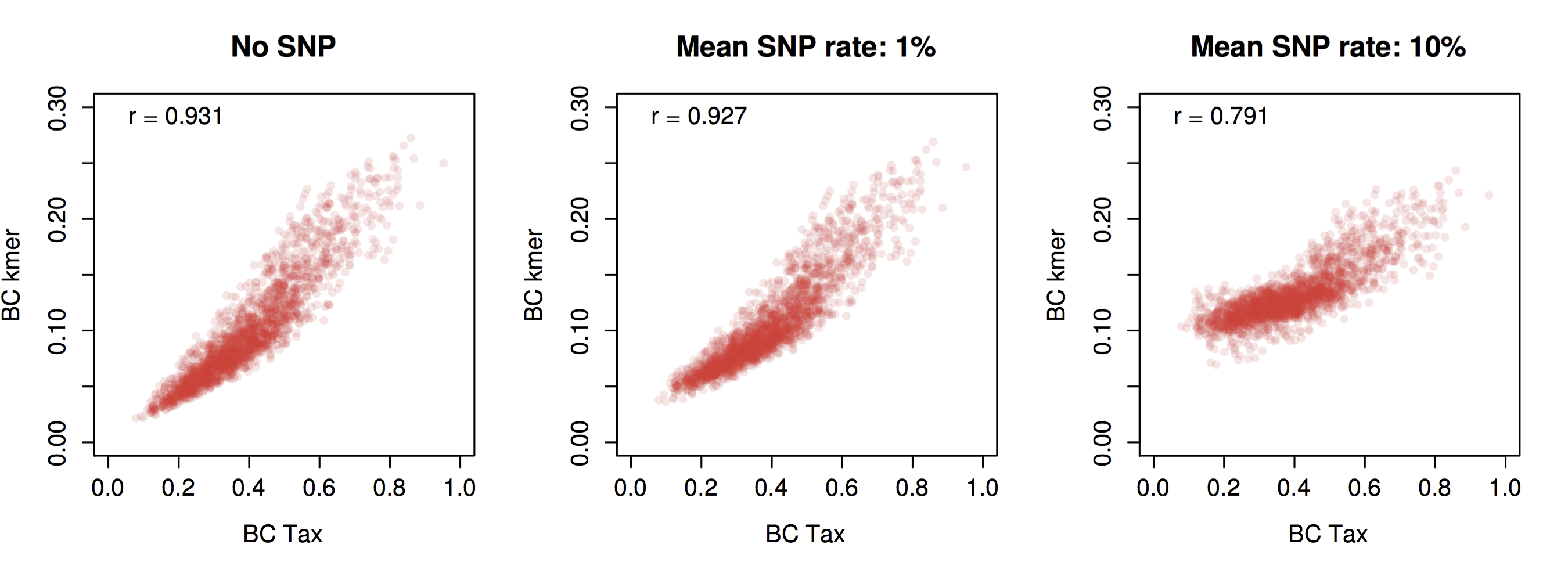

Supplement: Additional file 3 — Figure S1. Comparison of pairwise difference measures obtained by k-mer and taxonomic composition (50 simulated metagenomes). For each pair Y-axis represents k-mer dissimilarity, X-axis represents dissimilarity by taxonomic composition. k=10 A) using the original bacterial genomes; B) using the bacterial genomes with 1 % of SNPs. (PNG 451 kb) [file 12859_2015_875_MOESM3_ESM.png]

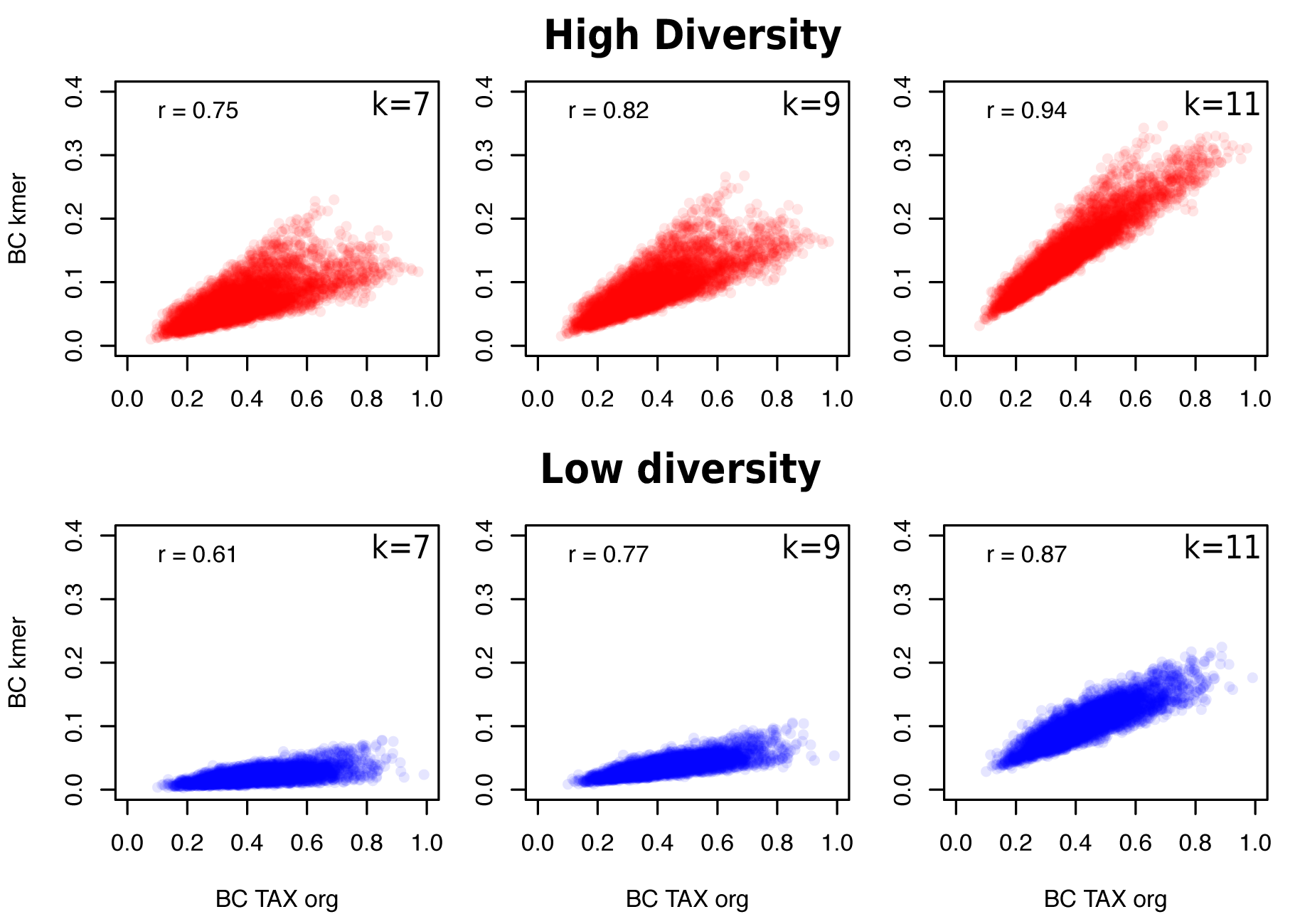

Supplement: Additional file 4 — Figure S2. Comparison of pairwise dissimilarity measures obtained by k-mer and taxonomic composition for simulated for high- and low-diversity metagenomes. As seen, satisfactory correlation of k-mers with taxonomic composition can be obtained only at relatively high values of k. (PNG 233 kb) [file 12859_2015_875_MOESM4_ESM.png]

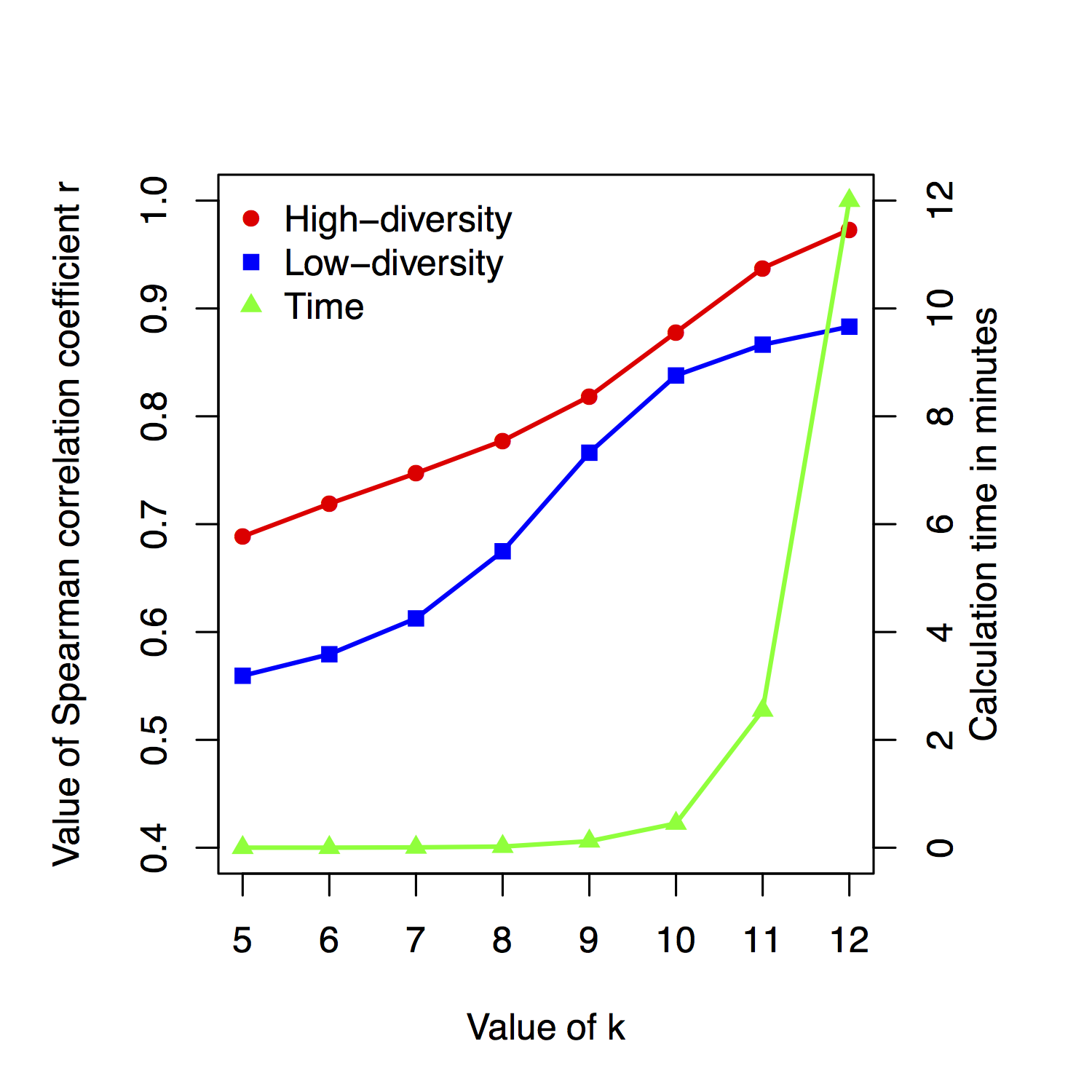

Supplement: Additional file 5 — Figure S3. Correlation between k-mer and taxonomic composition dissimilarity matrices, as well as k-mer dissimilarity matrix computation time with varying values of k. All computations were performed on a compute node with CPU Opteron 6176 2.3 GHz (24 cores) and 64 Gb RAM. (PNG 185 kb) [file 12859_2015_875_MOESM5_ESM.png]

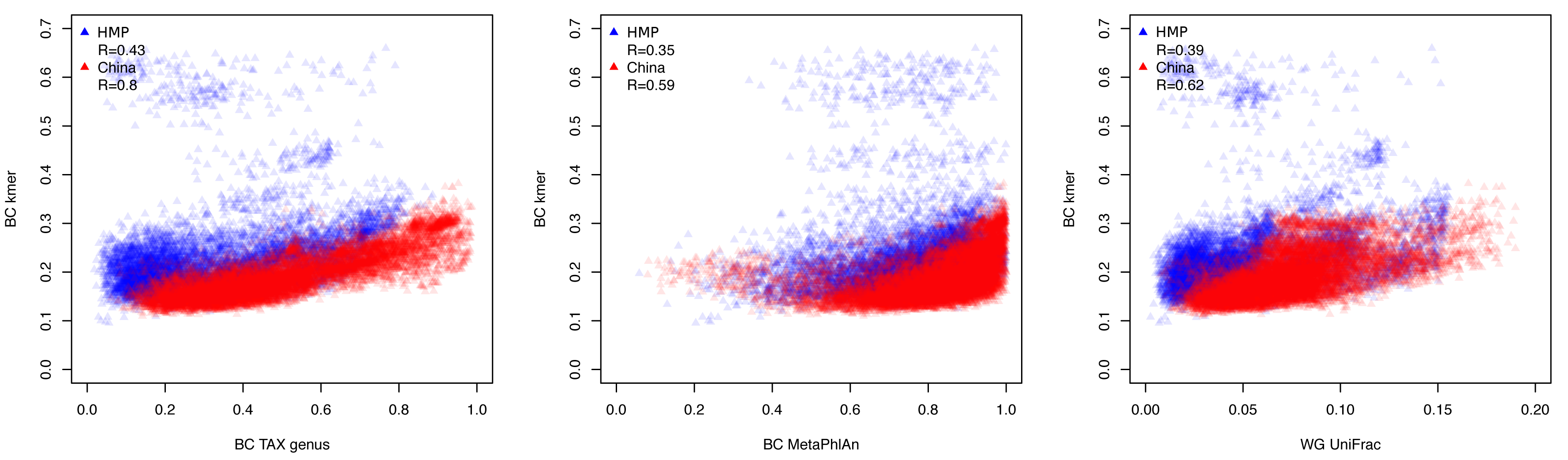

Supplement: Additional file 6 — Figure S4. Comparison of dissimilarity measures obtained by k-mer and 3 reference-based methods: BC MetaPhlAn genus, BC MetaPhlAn org and WG UniFrac. For each plot, Y-axis represents k-mer dissimilarity, X-axis - dissimilarity using one of reference-based methods. (PNG 925 kb) [file 12859_2015_875_MOESM6_ESM.png]
